# DGLA from the Microalga *Lobosphaera Incsa* P127 Modulates Inflammatory Response, Inhibits *iNOS* Expression and Alleviates NO Secretion in RAW264.7 Murine Macrophages

**DOI:** 10.3390/nu12092892

**Published:** 2020-09-22

**Authors:** Ekaterina Novichkova, Katya Chumin, Noy Eretz-Kdosha, Sammy Boussiba, Jacob Gopas, Guy Cohen, Inna Khozin-Goldberg

**Affiliations:** 1 Microalgal Biotechnology Laboratory, The French Associates Institute for Agriculture and Biotechnology for Drylands, The Jacob Blaustein Institutes for Desert Research, Ben-Gurion University of the Negev, Midreshet Ben-Gurion 8499000, Israel; novichko@post.bgu.ac.il (E.N.); sammy@bgu.ac.il (S.B.); 2 The Albert Katz International School for Desert Studies, The Jacob Blaustein Institutes for Desert Research, Ben-Gurion University of the Negev, Midreshet Ben-Gurion 8499000, Israel; 3 The Skin Research Institute, The Dead-Sea and Arava Science Centre, Masada 86910, Israel; katya@adssc.org (K.C.); noy@adssc.org (N.E.-K.); guy@adssc.org (G.C.); 4 Department of Microbiology and Immunology and Genetics, Faculty of Health Sciences, Ben-Gurion University of the Negev, Beer Sheva 8400501, Israel; jacob@bgu.ac.il; 5 Department of Oncology, Soroka University Medical Center, Beer Sheva 8400501, Israel; 6 Eilat Campus, Ben-Gurion University of the Negev, Eilat 8855630, Israel

**Keywords:** dihomo-γ-linolenic acid, microalgal biotechnology, prostaglandin E1, nitric oxide, immunomodulation

## Abstract

Microalgae have been considered as a renewable source of nutritional, cosmetic and pharmaceutical compounds. The ability to produce health-beneficial long-chain polyunsaturated fatty acids (LC-PUFA) is of high interest. LC-PUFA and their metabolic lipid mediators, modulate key inflammatory pathways in numerous models. In particular, the metabolism of arachidonic acid under inflammatory challenge influences the immune reactivity of macrophages. However, less is known about another omega-6 LC-PUFA, dihomo-γ-linolenic acid (DGLA), which exhibits potent anti-inflammatory activities, which contrast with its delta-5 desaturase product, arachidonic acid (ARA). In this work, we examined whether administrating DGLA would modulate the inflammatory response in the RAW264.7 murine macrophage cell line. DGLA was applied for 24 h in the forms of carboxylic (free) acid, ethyl ester, and ethyl esters obtained from the DGLA-accumulating delta-5 desaturase mutant strain P127 of the green microalga *Lobosphaera incisa*. DGLA induced a dose-dependent increase in the RAW264.7 cells’ basal secretion of the prostaglandin PGE1. Upon bacterial lipopolysaccharide (LPS) stimuli, the enhanced production of pro-inflammatory cytokines, tumor necrosis factor alpha (TNFα) and interleukin 1β (IL-1β), was affected little by DGLA, while interleukin 6 (IL-6), nitric oxide, and total reactive oxygen species (ROS) decreased significantly. DGLA administered at 100 µM in all forms attenuated the LPS-induced expression of the key inflammatory genes in a concerted manner, in particular *iNOS*, *IL-6*, and *LxR*, in the form of free acid. PGE1 was the major prostaglandin detected in DGLA-supplemented culture supernatants, whose production prevailed over ARA-derived PGE2 and PGD2, which were less affected by LPS-stimulation compared with the vehicle control. An overall pattern of change indicated DGLA’s induced alleviation of the inflammatory state. Finally, our results indicate that microalgae-derived, DGLA-enriched ethyl esters (30%) exhibited similar activities to DGLA ethyl esters, strengthening the potential of this microalga as a potent source of this rare anti-inflammatory fatty acid.

## 1. Introduction

Long-chain polyunsaturated fatty acids (LC-PUFA) are key precursors of a broad array of lipid mediators of health and disease. LC-PUFA of the omega-3 family (e.g., eicosapentanoic acid, EPA, 20:5 *n*-3, docosahexaenoic acid, 22:6 *n*-3) and the omega-6 family (e.g., arachidonic acid, ARA, 20:4 *n*-6) have attracted considerable attention because of their numerous biomedical activities, and the important roles they play in human health and nutrition [1]. The immediate precursor of ARA, dihomo-γ-linolenic acid (DGLA), is a 20-carbon omega-6 LC-PUFA (20:3 *n*-6), derived in vivo from the essential omega-6 linoleic acid (18:2 *n*-6). DGLA has recently emerged as an anti-inflammatory, anti-proliferative and anti-atherogenic LC-PUFA [2,3,4,5,6]. The health-beneficial properties of DGLA are related to its crosstalk with ARA metabolism and the production of a distinct group of eicosanoids and other lipid mediators that contribute to the resolution of inflammation and influence survival of cancer cells [6]. However, the natural sources of DGLA are very limited as it occurs as an intermediate in ARA biosynthesis and does not accumulate in considerable amounts [7].

Dietary DGLA is generally provided in the form of its precursor γ-linolenic acid (GLA, 18:3 *n*-6), derived from rare botanical oils or genetically-modified organisms [8]. To exert its biological and pharmaceutical activities in the human body, dietary GLA is initially elongated to DGLA. Thus, compared with its precursor GLA, the dietary DGLA bypasses not only the Δ6 desaturase but also an elongase, required for DGLA biosynthesis from LA. Since the activity of enzymes involved in LC-PUFA biosynthesis is impaired with aging and disease [9], dietary DGLA is advantageous over GLA. The effects of DGLA were also studied through pharmaceutical inhibition [10] and genetic manipulation of the delta-5 desaturase that catalyzes the conversion of DGLA to ARA [11]. Similarly, several single-celled ARA-accumulating oleaginous organisms, such as microscopic fungi and microalgae, can be manipulated to produce DGLA through the inactivation of the delta-5 desaturase. Likewise, the delta-5 desaturase mutants were produced in the oleaginous fungus *Mortierella* [12,13], and the green microalga *Lobosphaera incisa* (Trebouxiophyceae, Chlorophyta) [7].

The ratio of omega 3 and omega 6 PUFA governs the array of lipid mediator generation from them and thus influences the outcomes of various human conditions with inflammatory components. In particular, the fatty acid composition of inflammatory and immune cells significantly changes with the manipulation of LC-PUFA uptake [1]. For instance, ARA supplementation had been shown to increase interleukin 6 (IL-6) by 3-5 fold in a prostacyclin-dependent manner [14]. Conversely, omega 3 LC-PUFA, EPA and docosahexaenoic acid (DHA), had been shown to reduce IL-6 and tumor necrosis factor alfa (TNFα) secretion in RAW264.7 macrophages [15]. The precursor LC-PUFA determines the type of prostanoids, of series 1, 2, or 3, that are produced from DGLA, ARA, and EPA, respectively [16]. Metabolism of ARA generates a myriad of oxylipins, prostanoids and eicosanoids, formed via cyclooxygenase (COX), lipoxygenase (LOX), and CYP routes [17], and peroxidation products [18,19], which are generally considered proinflammatory. However, certain ARA metabolites play important roles in the resolution of inflammation [20]. DGLA-derived prostaglandin E1 (PGE1) and lipoxygenase (LOX-15) products 15-(S)-hydroxy-8,11,13-eicosatrienoic acid (15-HETrE) are considered anti-inflammatory molecules. Lacking the Δ5 double bond, DGLA cannot serve as a substrate for the LOX-5-mediated formation of pro-inflammatory leukotriene LTB4; moreover, DGLA-derived LOX15 products inhibits LTB4 production [4]. Furthermore, 8-hydroxyoctanoic acid, formed from COX-2-catalyzed DGLA peroxidation, suppresses colon cancer cell proliferation [11].

Despite the current progress, the range of DGLA activities is still not sufficiently studied, in part due to its limited natural sources. Single-cell organisms, such as marine microalgae, some fungi, and related microorganisms, are the primary producers of LC-PUFA. Microalgae are regarded as renewable producers of functional ingredients for both animal and human food that can be cultivated under controlled conditions, and they can produce dietary ingredients using light, CO_2_ and mineral nutrients. However, despite their high biotechnological potential, only a limited number of photosynthetic species are currently cultivated mainly for high-value nutraceuticals, such as lucrative carotenoids and omega-3 LC-PUFA (EPA) [21]. The fresh microalga *L. incisa* is exceptionally rich in omega-6 ARA as it accumulates triacylglycerols with high ARA proportions under nitrogen starvation conditions [22]. This ability is rarely encountered in microalgal lineages and absent from land plants. The delta-5 desaturase mutant of *L. incisa* P127, deficient in ARA, represents a prospective oleaginous organism for the photosynthesis-driven biotechnological production of DGLA for dietary and medicinal applications [7,23]. It accumulates up to 30% of DGLA in storage lipids, triacylglycerols, under nitrogen starvation conditions [24].

In this work, we aimed to investigate the immunomodulatory effects of DGLA in RAW264.7 murine macrophage cells, administered in different forms, including the *L. incisa* P127-derived DGLA. We showed that endogenous DGLA might change the basal secretion levels of the prostaglandins PGE1 and PGE2. DGLA suppressed the bacterial lipopolysaccharide (LPS)-induced NO and cytokine IL-6 secretion and the expression of several genes implicated in the inflammatory response. Overproduction of PGE1 was associated with less affected PGE2 and PGD2 levels coupled with a coordinated shift in pro-inflammatory gene expression, suggesting alleviation of the inflammatory state upon DGLA treatment.

## 2. Materials and Methods

### 2.1. Materials

Biological Industries (Beit Haemek, Israel) supplied all cell culturing compounds and tetrazolium-formazan (XTT) assay kit. Cytokine Elisa assays were obtained from Bioligand (San Diego, CA, USA). PGD2 assays were from Cayman Chemicals (Ann Arbor, MI, USA) and PGE1 and PGE2 from Enzo (Lörrach, Germany); 5-(and 6) -carboxy-2’ 7’-dichlorodihydrofluorescein diacetate (DC-FDA) was obtained from Invitrogen Molecular Probes (Eugene, OR, USA). Unless specified, reagents and analytical grade substrates were obtained from Sigma-Aldrich (Rehovot, Israel). Free ethyl ester and DGLA were from Nu-Chek Prep Inc. (Elysian, MN, USA).

### 2.2. Microalga Cultivation and Direct Ethylation of Biomass

The cultures of *L. incisa* mutant strain P127 were grown in 1-L glass columns bubbled with 1.5% CO_2_ in nutrient-replete mBG11 medium for 3 d. Cells were harvested by centrifugation and resuspended at a biomass density of 1 mg mL^−1^ in an *n*-depleted mBG11 medium to induce N starvation. Cultures were then cultivated under a light intensity of 170 μmol photons m^−2^ s^−1^ for 14 d [24]. The fatty acid profile and dry weight content were regularly examined. After reaching a DGLA content of ~10% of biomass (~30% DGLA of total fatty acids), the biomass was harvested by centrifugation, and freeze-dried. Ethyl esters of total cellular fatty acids were obtained on 50 mg aliquots following incubation in 2 mL of 2% H_2_SO_4_ in absolute ethanol (v/v) at 85 °C for 1.5 h. The reaction was terminated by the addition of double distilled water (DDW), and fatty acid ethyl esters were recovered by several sequential extractions with hexane, dried under nitrogen gas flow, and weighed. For DGLA quantification, a small fraction was injected into a gas chromatography with flame ionization detector (GC-FID) after the addition of an internal standard.

### 2.3. Cell Culture and Treatments

RAW 264.7 murine macrophage cell line was obtained from the American Type Culture Collection (Rockville, MD, USA). Cells were maintained in a Dulbecco’s modified Eagle’s medium (DMEM) high-glucose medium supplemented with 10% fetal bovine serum (FBS), 2 mM L-glutamine, and 1% penicillin/streptomycin and incubated at 5% CO_2_ at 37 °C in a humidified incubator. For routine maintenance and experimental seedings, the cells were detached by a cell scraper and counted with a hemocytometer.

For DGLA treatments, RAW 264.7 cells were plated at 4 × 10^5^ cell/mL in complete growth medium (100 µL in a 96-well plate and 1 mL in a 12-well format). Cells were incubated for 24 h with DGLA-free acid (Nu-Chek Prep, Elysian, MN, USA) DGLA ethyl ester (Nu-Chek Prep), or DGLA-rich ethyl esters obtained from P127 biomass. Stock solutions were prepared in dimethyl sulfoxide (DMSO), such that the final DMSO concentration in the cell culture media was set at 0.1% in all treatment groups. Where indicated, cells were induced with 100 ng/mL of LPS (*E. coli*, Sigma). After 24 h, cells were collected by scraping for fatty acid analysis and RNA extraction. Culture supernatants were collected for quantification of NO, cytokine, and prostaglandin secretion.

### 2.4. Cell Viability

Following treatments, RAW 264.7 cells viability was determined by 3-(4,5- dimethylthiazol-2-yl)-2,5-diphenyltetrazolium bromide (MTT) and XTT assays. Briefly, cells were incubated with MTT (0.5 mg/mL) in PBS for 1 h at 37 °C. The medium was then aspirated, and isopropanol was added to solubilize the colored crystals. Absorbance at 570 nm was measured in a plate reader. XTT was performed according to the manufacturer’s instructions with a 4-h incubation period. In addition, a trypan blue exclusion assay and cell counts were used in order to exclude any impact of the different treatments on the colorimetric assay.

### 2.5. Lipid Extraction

To determine DGLA incorporation into membrane lipids, cells were harvested, washed with phosphate-buffered saline (PBS), and extracted according to the method of Bligh and Dyer 1959 [25]. Neutral and polar lipids were separated by silica-gel chromatography on 500 Bond Elut cartridges (Agilent), using chloroform and methanol for elution, respectively. Fatty acid composition of polar lipids was determined by GC-FID.

### 2.6. Fatty Acid Analysis by Gas Chromatography with Flame Ionization Detector (GC-FID)

To analyze the fatty composition of RAW 264.7 cells and algal biomass, transmethylation of fatty acids was performed by incubating polar lipids or freeze-dried biomass in dry methanol containing 2% (v/v) H2SO4 at 80 °C for 1.5 h under an argon atmosphere and continuous stirring. Pentanoic acid (C15:0; Sigma–Aldrich) was added as an internal standard. FAMEs were quantified on a Trace GC ultra (Thermo, Italy) equipped with a flame ionization detector and a programmed temperature vaporizing (PTV) injector. The detector temperature was fixed at 280 °C, and helium was used as a carrier gas. The PTV injector was programmed to increase the temperature from 40 °C at the time of injection to 300 °C at time of sample transfer. Separation was achieved on a fused silica capillary column (Supelco-WAX, 30 m × 0.32 mm). FAMEs were identified by co-chromatography with authentic standards (Sigma–Aldrich, Rehovot, Israel) [26].

### 2.7. Interleukin 6(IL-6), Nitric Oxid, Prostaglandin E1, E2 and D2 Quantification

Following treatment, the spent medium was collected, cleared by centrifugation (Labofuge 400R, 1550 rpm, 5 min.), aliquoted and stored at −80 °C until used. IL-6, PGE1, PGE2, and PGD2 quantification was performed by enzyme-linked immunosorbent assay (ELISA) kits, according to the manufacturer’s instructions and using standard curves. Nitric oxide (NO) secretion was determined in freshly collected medium aliquots using the Griess assay, which detects nitrite formed from NO conversion in the medium [27]. Briefly, the spent medium was mixed with the Greiss reagent in equal volumes, and the absorbance was measured at the wavelength 540 nm using a microplate reader (Infinite f200, TECAN). Sodium nitrite was used for calibration curve generation.

### 2.8. Determination of Intracellular Reactive Oxygen Species (ROS)

The cells were treated with the indicated DGLA concentrations with or without LPS for 24 h. Then, the cells were mounted with 50 µM of the green fluorescence dye 5-(and 6) -carboxy-2’ 7’-dichlorodihydrofluorescein diacetate (DC-FDA) for 30 min, and fluorescence was determined (ex. 485 nm, em. 538 nm). Blank (cells not mounted with the dye) readings were subtracted from all treatments.

### 2.9. RNA Isolation, cDNA Preparation and Real-Time Polymerase Chain Reaction (PCR) Analysis of Gene Expression

RNA extraction from pelleted and washed cells was carried out using a SV Total RNA Isolation kit (Promega, Madison, WI, USA) following the manufacturer’s instructions. Total RNA was quantified by a Nanodrop spectrophotometer (Thermo Scientific, Waltham, MA, USA), and cDNA synthesis was performed using a qPCRBIO High quality cDNA synthesis kit (Thermo Scientific) following the manufacturer’s instructions. A quantitative real-time polymerase chain reaction (qRT-PCR) was performed on several target genes (Table S1) on a CFX96 Cycler (Bio-Rad) using an iTaq Universal SYBR Green Supermix (Bio-Rad). Primers used in this study are presented in Table S1. Three technical replicates from each of the four biological replicates were used. Data were analyzed using the comparative ^ΔΔ^ Ct method. Results were normalized to the expression of a housekeeping gene hypoxanthine phosphoribosyltransferase 1 (HPRT1).

### 2.10. Statistical Analysis

Statistical analyses were performed using a single factor analysis of variance (ANOVA); *n* = 3; *p* < 0.05 was considered significant.

## 3. Results

### 3.1. Dihomo-γ-Linolenic Acid (DGLA) Induces Prostaglandin Secretion in RAW264.7 Macrophage Cells

**Figure 1:**
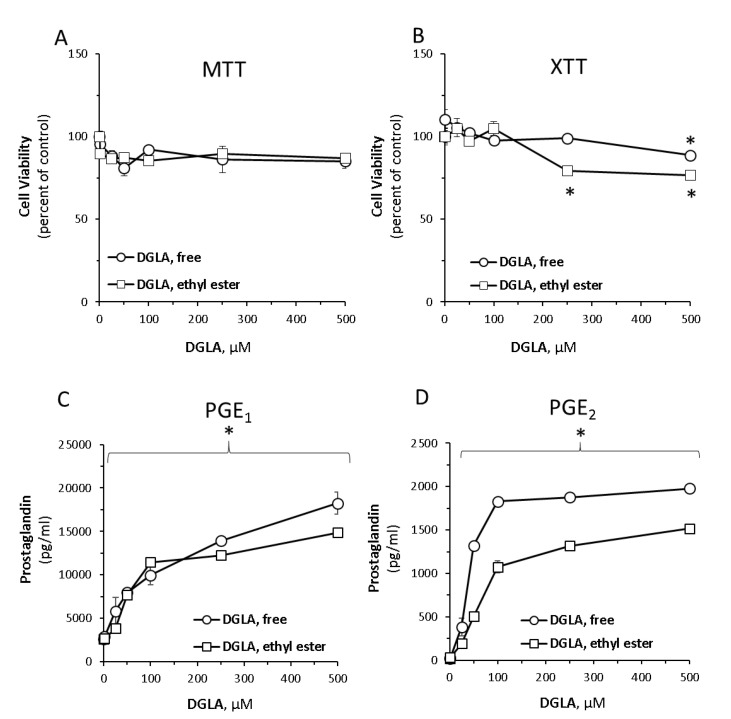
Dose-dependent response evaluation of dihomo-γ-linolenic acid (DGLA) free acid and ethyl ester forms in RAW264.7 macrophages. Cells were treated with increasing concentrations of DGLA in its free or ethyl ester form for 24 h. Cell viability was determined by 3-(4,5- dimethylthiazol-2-yl)-2,5-diphenyltetrazolium bromide (MTT) (**A**) and tetrazolium-formazan (XTT) (**B**) assays, as indicated in the Methods section. Prostaglandin E1 (PGE1) (**C**) and prostaglandin E2 (PGE2) (**D**) were quantified in culture supernatants. Dimethyl sulfoxide (DMSO) at 1:1000 (the final concentration in the cell culture media was 0.1% in all treatment groups) was used as a vehicle control and did not affect the cells viability. Data are presented as mean ± standard deviation (SD). * denotes a significant difference compared to untreated control, *p* < 0.05, *n* = 3.

To first evaluate the impact of DGLA on RAW264.7 murine macrophage cells, dose-response analyses were performed on two DGLA preparations—free carboxylic acid and esterified (ethyl ester) DGLA forms. To evaluate the impact of DGLA on cell viability, both MTT and XTT were used. Collectively, the results indicated that the two compounds were well tolerated by the cells up-to 250 µM (Figure 1A,B). At higher concentrations, some reduction in cell viability was noticeable, in particular when examining the XTT results of the ethyl ester form.

Concomitantly, the impact of DGLA treatment was evaluated on several known immunomodulators, such as the prostaglandins PGE1 and PGE2, and key cytokines in un-stimulated RAW264.7 cells. As shown in Figure 1C,D, PGE1 and PGE2 levels dose-dependently increased in the media over 100-fold. In particular, a massive increase PGE1 indicates for the predominant conversion of DGLA to PGE1. Distinctly from prostaglandins, basal levels of nitric oxide, TNFα, IL-1β and IL-6 detected in the culture supernatants were not significantly altered by the DGLA treatments (Figure S1).

### 3.2. DGLA Supplementation Modulates Bacterial Lipopolysaccharide (LPS)-Induced Inflammation in RAW264.7 Macrophages

**Figure 2:**
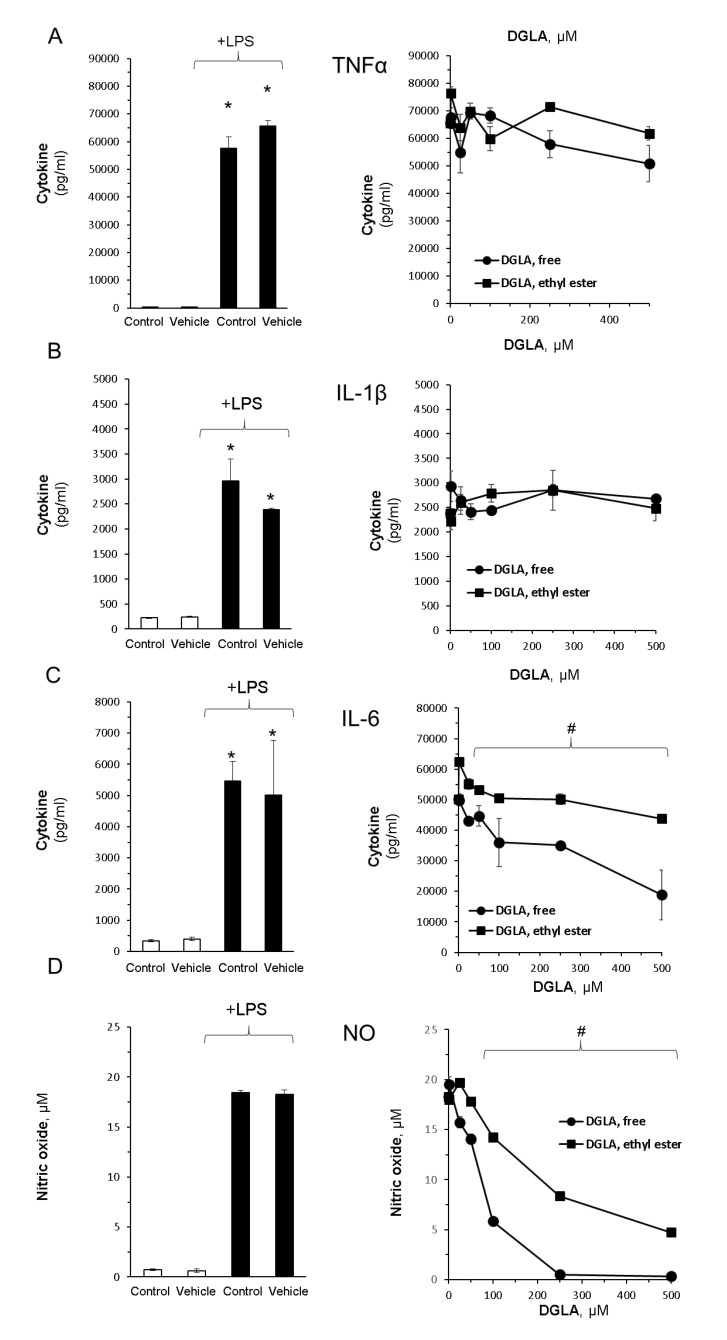
DGLA modulates key inflammatory signals in bacterial lipopolysaccharide (LPS)-stimulated RAW264.7 macrophages. Inflammation was induced by LPS (100 ng/mL). Concomitantly, the cells were treated without or with increasing DGLA concentrations. DGLA was administered as free acid or ethyl ester form for 24 h. Tumor necrosis factor α (TNFα; (**A**)), interleukin 1β (IL-1β; (**B**)); interleukin 6 (IL-6; (**C**)), nitric oxide (NO; (**D**)), and were quantified in culture supernatants after 24 h. Data are presented as mean ± SD. *^/#^ denotes a significant difference compared to untreated control or LPS-stimulated control, respectively, *p* < 0.05, *n* = 3. Control—untreated cells; vehicle—cells treated with 0.1% DMSO, the solvent used in DGLA preparations. The left panel depicts the impact of LPS stimuli and lack of effect DMSO, whereas the right panel depicts the impact of increasing concentrations of DGLA on LPS-stimulated cells.

In order to evaluate DGLA’ influence on inflammatory cascades, the RAW264.7 macrophages were stimulated with LPS in the absence or presence of the free acid and ethyl ester DGLA forms. As shown in Figure 2, the levels of proinflammatory cytokines TNFα and IL-1β were markedly enhanced following LPS induction but were unaltered by DGLA (Figure 2A,B). In contrast, IL-6 showed a gradual and significant reduction upon DGLA administration (Figure 2C).

**Figure 3:**
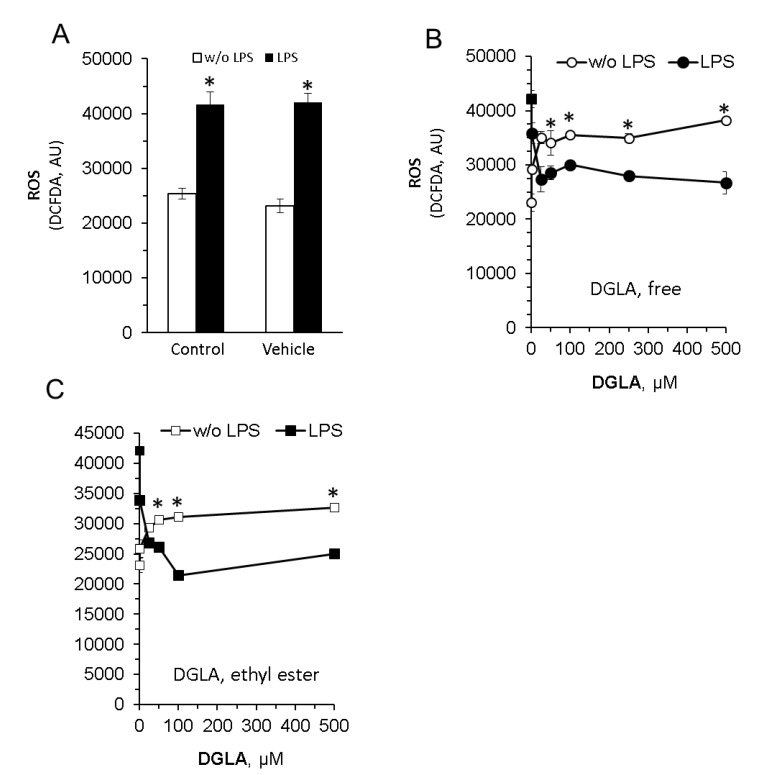
DGLA alters reactive oxygen species (ROS) production in RAW264.7 macrophages. Inflammation in the cells was induced by LPS (100 ng/mL). Concomitantly, the cells were treated without (w/o) or with increasing DGLA concentrations. After 24 h of incubation, cells were mounted with a fluorogenic dye 5-(and 6) -carboxy-2’ 7’-dichlorodihydrofluorescein diacetate (DC-FDA), and ROS levels were estimated by fluorescence measurements. ROS levels, enhanced by LPS (**A**) or modulated by DGLA administered as free acid or ethyl ester form (**B**,**C**), are presented. Data are presented as mean ± SD. * denotes a significant difference compared to respective control, *p* < 0.05, *n* = 3. Control—untreated cells; vehicle—cells treated with 0.1% DMSO, the solvent used in DGLA preparations.

RAW264.7 macrophages produce NO in response to bacterial endotoxin stimulation (Figure 2B, left panel). Importantly, the nitric oxide (NO) levels determined in the culture supernatants were dramatically attenuated by DGLA in a dose-dependent manner (Figure 2D, right panel), suggesting that NO generation is tightly regulated by its cellular level. Of note, both the efficacy and potency of free DGLA were higher compared with the ethyl ester form. Since increased oxidative stress and ROS generation have been linked to inflammatory processes in numerous cell types, we next determined cellular ROS generation using the DC-FDA method. The results presented in Figure 3 show a differential impact of DGLA, inducing ROS generation without LPS stimuli, while reducing ROS levels with LPS stimuli.

**Figure 4:**
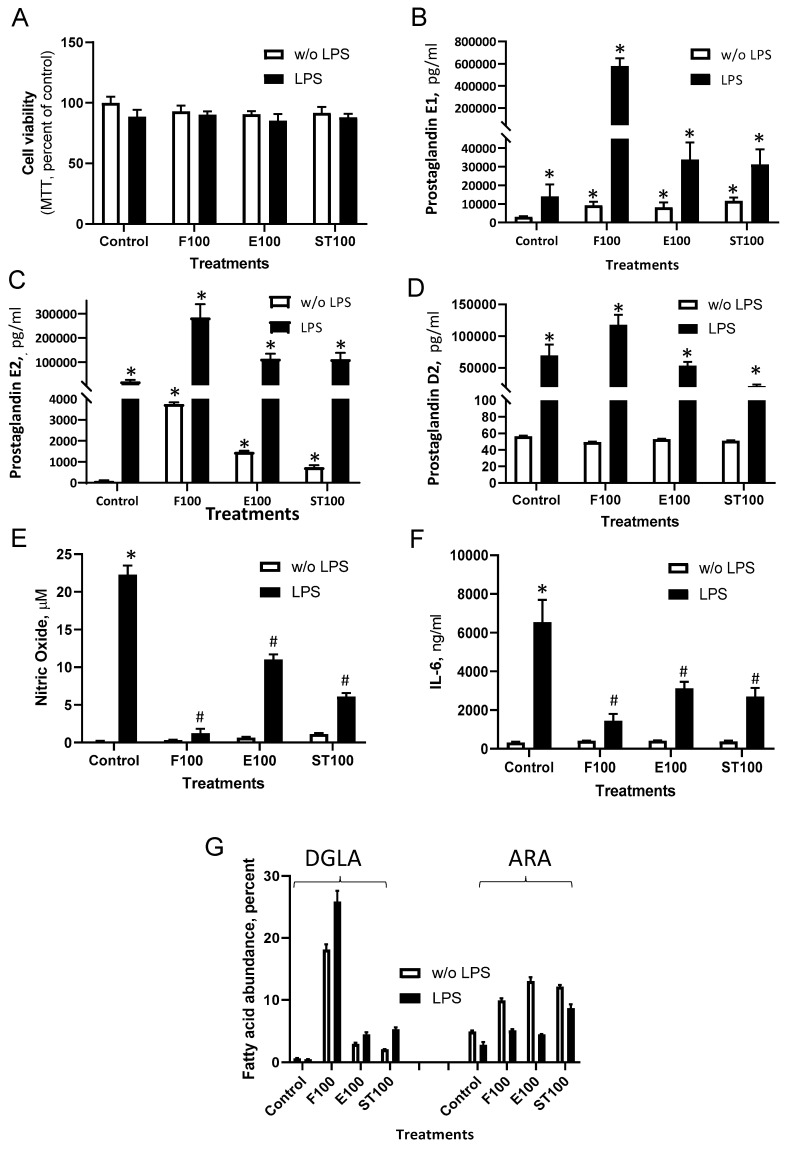
Synthetic and algae-derived DGLA modulate inflammation signals in RAW264.7 macrophages. Cells were treated as indicated in the legend of Figure 3. After 24 h, cell viability (**A**), PGE1, PGE2, and PGD2 (**B**–**D**), NO (**E**), and IL-6 (**F**) were quantified in culture supernatants. DGLA and arachidonic acid (ARA) levels in polar lipids (% of fatty acids) are presented in (**G**). Data are presented as mean ± SD. *^/#^ denote a significant difference compared to control (DMSO-treated) or LPS-induced cells, respectively, *p* < 0.05, *n* = 4. DGLA was administrated at 100 µM in three forms: Free acid (F100), Ethyl esters (E100) and microalgae-derived ethyl esters (ST100), respectively.

To further evaluate DGLA activity and reveal the potential of the alternative natural source, the use of microalga-based preparations was examined comparatively with synthetic sources. A DGLA-enriched (~30%) ethyl ester fraction (St) was obtained from nitrogen-starved *L. incisa* P127 cells as described in the Materials and Methods section. A concentration of 100 µM DGLA was selected for all treatments for further experiments as non-toxic (Figure 4A) and more physiologically relevant. As expected, LPS significantly enhanced PGE1 and PGE2 and PGD2 secretion levels (Figure 4B–D). Importantly, both the basal and LPS-stimulated levels of PGE1 and PGE2 increased in culture supernatants upon DGLA treatment. In particular, the ratio (stimulated/non-stimulated cells) of PGE1 enhancement upon DGLA treatment was significantly higher than that of PGE2 [41 folds vs. 13.7] indicating a shift in prostaglandin synthesis towards the anti-inflammatory PGE1. Moreover, the basal and stimulatory levels of PGD2 were almost unaffected by DGLA. In addition, massive reductions in NO and in IL-6 were detected (Figure 4E,F), in particular in the cells supplemented with DGLA free acid, which displayed the highest level of DGLA incorporation into polar lipids (Figure 4G, Figure S2). It is notable that the microalgae-derived ethyl esters, composed of four major fatty acids (16:0, 18:1, 18:2, DGLA) (Figure S3), exhibited similar activity to synthetic DGLA preparations, administrated at the equivalent DGLA levels.

### 3.3. DGLA-Mediated Modulation of Gene Expression

**Figure 5:**
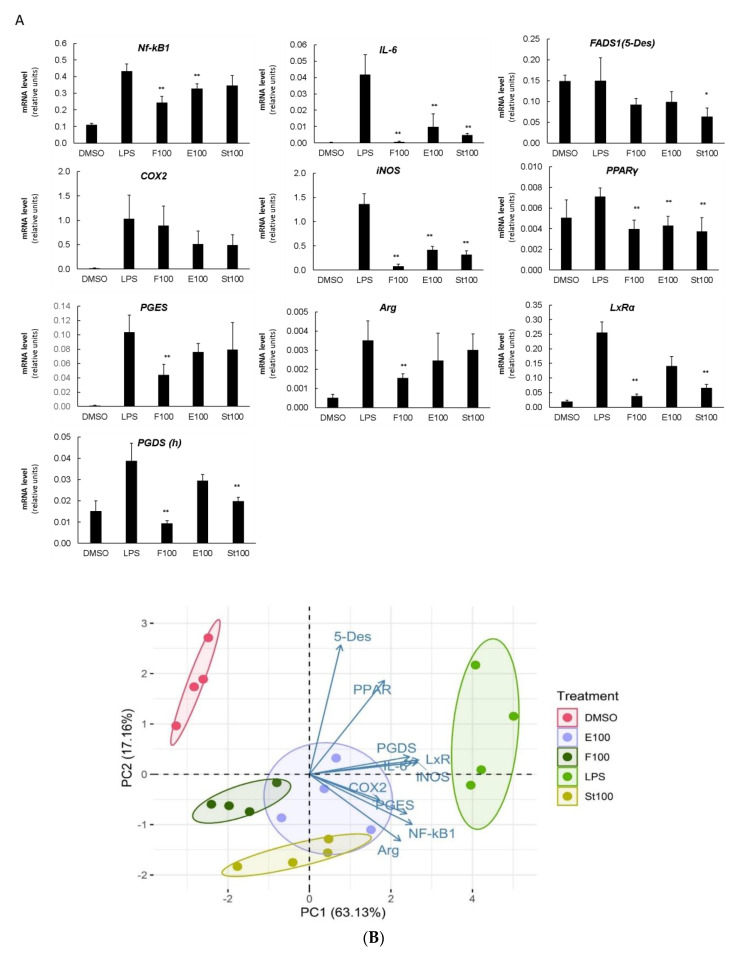
DGLA coordinately modulates the expression of key inflammatory and fatty acid metabolism-related genes in LPS-induced RAW264.7 macrophages. (**A**). Relative normalized expression (**B**). Principal component analysis was performed in R integrated package (four biological replicates are shown as individual circles). Cells were treated as indicated in the legend of Figure 3. mRNA was quantified by quantitative real-time polymerase chain reaction (qRT-PCR). Data are presented as mean ± SD. * and ** denote a significant difference compared to the LPS-induced vehicle control, *p* < 0.05, and *p* < 0.001, respectively, *n* = 4.

To evaluate the mechanism of immunomodulatory action, the DGLA’s effects on the expression of several target genes, representing key pathways implicated in the inflammatory cascade and LC-PUFA metabolism (Table S1), were determined by quantitative real-time PCR. In LPS-induced cells, DGLA in all forms significantly modulated the expression levels of the majority of examined genes. All genes, except *FADS1* (encoding the delta-5 desaturase, 5-Des) and *PPARγ*, were strongly induced upon LPS stimulation (Figure 5A). The activation of the NF-kB transcription factor is required to induce the expression of *COX-2* in LPS-stimulated RAW 264.7 cells [28]. Upon DGLA treatment, the LPS-induced levels of Nf-kB1 subunit expression decreased with free DGLA (by ~45%) followed by ethyl ester (by 15%). *COX2* and *FADS1* (5-Des) transcripts showed a decreasing tendency. Downstream of COX2, prostaglandin E synthase (*PGES*), and prostaglandin D synthase (*PGDS*) transcripts also decreased with free DGLA and algal DGLA-rich ethyl esters (specifically, *PGDS*). Importantly, DGLA strongly and coordinately attenuated the LPS-induced expression of both inducible nitric oxide synthase (iNOS) and IL6 genes. The LxRα (liver × receptor alfa) gene displayed a pattern of expression similar to *iNOS* and *IL-6*. The transcripts for M2 polarization-associated markers, arginase (*Arg*), and transcriptional factor PPARγ, were also reduced.

A principal component analysis (PCA) performed on the qRT-PCR results demonstrated that the high level of variation in gene expression between the vehicle control, DGLA-treatments and LPS-induced control cells can be explained by the first component (PC1, 63,1%) (Figure 5B). Cells treated with DGLA in all forms differed significantly from both the vehicle control and LPS-induced cells. Results revealed a clear separation between the control groups (DMSO, and LPS) and the DGLA-supplemented groups, which all closely clustered on the plot. The contribution of each variable (gene expression) to the treatment effects is indicated by the variable factor map, where the length of the vector represents the gene’s contribution to the overall effect. The vectors for *PGDS*, *iNOS*, *LxRα*, *IL6* genes and *COX2*, *PGES*, *NF-kB1*, and *Arg* genes displayed a shared pattern on the vector map, suggesting their coordinated changes upon DGLA treatment, and the main contribution to the dispersion of samples on PC1.

## 4. Discussion

In this work, the impacts of omega-6 LC-PUFA DGLA and the DGLA-enriched ethyl esters, obtained from the mutant strain of *L. incisa*, were investigated in the RAW264.7 murine macrophage cell line, a well-established screening tool for investigating of immunomodulators. DGLA, provided in three forms, modulated RAW264.7 cell basal and inflammatory reactions upon LPS stimuli. Free, non-esterified DGLA imposed stronger effects on IL-6, NO and PGE1 production compared with the DGLA and microalga-derived ethyl esters. Since free DGLA was more efficiently incorporated into membrane lipids, it displayed better availability for conversion to anti-inflammatory PGs with and without LPS-stimulation. To our knowledge, this is first study to elucidate the anti-inflammatory properties of DGLA in the ethyl ester form. Ethyl esters have several advantages as a dietary and pharmaceutical form of fatty acid administration. For example, ethyl esters of omega-3 fatty acids are approved as prescription drugs to reduce high TAG blood levels [29]. In addition, ethyl esters are readily soluble in aqueous milieu, and produce fewer toxic effects than non-esterified free acids. Furthermore, DGLA-enriched ethyl esters can be relatively easily obtained by direct transethylation of microalgal biomass. Importantly, the microalgae-derived ethyl esters exhibited similar activity to DGLA ethyl esters despite the presence of additional more common fatty acids. *L. incisa* P127-derived ethyl esters had a lower efficacy than the free form of DGLA. However, this form can be more feasible to produce as a commercial health-promoting agent, since the production of ethyl esters allows avoiding expenditures associated with extraction and simplifies the complex processing steps of microalgal biomass valorization.

RAW264.7 macrophage cells produce pro-inflammatory cytokines TNFα and IL-6, as well as release NO upon activation with bacterial endotoxin LPS—a classic response of inflammatory macrophages. TLR4-primed RAW264.7 cells upregulate *COX2* and *mPGES-1*, and secrete 2-series PGs, such as PGE2 and PGD2 [30]. We speculate that DGLA may exert anti-inflammatory influences in RAW264.7 cells through the modulation of COX2-mediated PGE1 production, and the attenuation of IL-6 and NO generation via inhibition of iNOS activity. As discussed below, our findings collectively suggest that NO and PGs are the key regulatory effectors altered by DGLA in RAW264.7 macrophages. As both NO and PGs are regulatory mediators in several physiological and pathophysiological processes, the impact of DGLA should be elucidated in other cell types. For instance, it can be foreseen that the dramatic reduction in NO would modulate the vascular system, including blood pressure regulation, and reduce platelet aggregation [31]. Enhanced PGE1 formation can be harnessed to treat erectile dysfunction [32]. It is clear the endothelial and vascular smooth muscle should be the target for future investigation.

DGLA-derived prostaglandins PGE1, PGD1 and 15-HETrE have been previously shown to be associated with anti-inflammatory effects in different models [3,10,33,34,35,36]. Our results on the enhancement of PGE1 production are therefore in line with previous publications. DGLA, applied in all three forms, stimulated the secretion of both PGE1 and PGE2 in the resting RAW264.7 cells, whereas PGD2 levels were unaffected. This may suggest a specific reaction and not an overall increase in prostaglandin production due to the elevated levels in the substrate. The formation of prostaglandins in unstimulated cells is likely mediated by the constitutive COX1, without triggering the signaling cascade, since none of the examined cytokines were elevated. PGE2 levels in non-induced and LPS-induced cells increased, suggesting the impact of DGLA on prostaglandin production in both environments.

Three major transcription factors (TFs) Nf-kB, PPARγ and LXRα, which participate in controlling inflammation, are tightly connected to fatty acid manipulations; all three TFs may influence the expression of iNOS and COX2 [37,38,39,40,41,42]. In agreement with the regulatory role of NF-κB on COX2 expression [43], mRNA levels for NF-kB1 and COX2 displayed a decreasing trend under LPS induction. Puzzlingly, the enhancement of PGE1 and PGE2 occurred despite a significant reduction in *PGES* mRNA. Similarly, the reduction in *PGDS* expression, which was most significant with DGLA administered as free acid, was not associated with the reduction in PGD2. This may indicate an autoregulatory action initiated by the high prostaglandin levels. Indeed, studies have previously reported that prostaglandins may regulate their biosynthesis gene expression [44,45,46]. From the other side, the attained transcriptional activation and concentration of precursors could be sufficient for prostaglandin generation. Interestingly, upon LPS stimuli, the levels of the three examined PGs were elevated, but to a different extent. Our study thus confirms DGLA’s role in controlling and regulating the PGE1/PGE2, and PGE1/PGD2 ratios. It is evident that not only the ratio between the anti-inflammatory to proinflammatory prostaglandins PGE1/PGE2, mediated by COX1 and COX2, is governed by availability of DGLA [6], but also the increased net production of anti-inflammatory prostaglandins PGE1 and PGD2, and possibly PGD1. Although the cellular ratio of DGLA/ARA may not be an effective route to enhance the endogenous synthesis of PGE1 over PGE2 in cells/tissues where COX-1 predominates over COX-2 [10], the exogenous DGLA effectively promoted PGE1 over PGE2 synthesis in RAW264.7 cells.

A strong inhibition of pro-inflammatory cytokine IL-6 with DGLA is in line with the alleviation of inflammation. Prostaglandins of pro-inflammatory (PGE2) and anti-inflammatory (PGE3) types may impact IL-6 production, generally by either inducing or not altering its production [47,48,49]. Administration of PGE1 was shown to reduce IL-6 production in inflammatory conditions [50,51]. Since 2-series prostaglandins were shown to induce IL-6 expression in LPS-activated RAW264.7 cells [49], we speculate that a significant reduction in IL-6 gene expression and production, upon DGLA treatment, may be mediated by the augmented levels of PGE1.

DGLA led to a massive reduction in NO production in RAW264.7 macrophages. DGLA also diminished intracellular production of ROS, but the effect on NO reduction was stronger. Activated macrophages produce NO via the inducible NOS to serve as a signaling molecule, effective vasodilator, and a potent antimicrobial agent; however, excessive NO generation may lead to cell/tissue damage and septic shock [52]. Reduction in NO production in activated macrophages evokes a plethora of consequences. The up-regulation of both *iNOS* and *COX-2* during inflammation is controlled by NF-κB [12], whereas a high intake of omega-3 fatty acids has been associated with reducing expression of COX2 [53,54] and iNOS [55]. As far as concerning effects of prostaglandins, specifically, PGE1 was shown to suppress the activity of NF-kB and reduce iNOS synthesis in primary human hepatocytes [56]. The effects of fatty acids of two families warrant further investigation in different cell types and with different types of prostaglandins.

PPARγ had been linked in PUFA regulation of gene expression, and its activation by omega-3 LC-PUFA is implicated in their anti-inflammatory action [57]. Our results showed that the mRNA levels of PPARγ decreased upon DGLA supplementation. ARA is known to regulate PPARγ expression; hence, it is plausible that an excess of omega-6 DGLA antagonized PPARγ expression. PPARγ is also implicated in the transcriptional regulation of LC-PUFA biosynthesis. At least with respect to gene expression, the effect of DGLA on PPARγ expression was found to be similar to that of *FADS1*, suggesting that the conversion of DGLA to ARA was affected. The functional crosstalk between prostaglandins, PGES and PPARγ at the onset of inflammation can be mediated through PGES-derived prostaglandin J2, which reduces the activity of PPARγ and the expression of COX2, and PGES, resulting in the reduction of PGE2 synthesis [58,59]. This can explain the common effect of DGLA administration on PPARγ, COX2 and PGES expression.

A number of other transcriptional factors are also regulated by fatty acids and lipids, such as Signal Transducer And Activator Of Transcription 1 (STAT1) and Liver X receptor α and β (LXRα and β). Macrophages synthesize anti-inflammatory fatty acids endogenously in an LXR-dependent manner, whereas LXRs is known to antagonize NFkB, and activates genes with anti-inflammatory activities [60]. However, in THP-1 human macrophages, DGLA’ effects on IFN-γ signaling were associated with a significant down-regulation of LXR-α and LXR-β expression along with modulation of the STAT1 serine 727 phosphorylation [3]. Also, the exogenous omega-3 EPA and LA inhibited LXR activity in RAW264.7 macrophages [61]. In our experiments, DGLA also drastically reduced mRNA levels of LXR-α to almost basal levels, in particular with the non-esterified DGLA. An inverse relationship between the exogenous DGLA and LXR expression levels suggests that DGLA’ effects in macrophages are independent of LxR activation or are regulated by a negative feedback loop mechanism. A strong and concerted repression of iNOS, IL-6 and LxR genes on DGLA treatment may suggest the common regulatory mechanism, which requires further study. Arginase (which competes with iNOS for the substrate arginine), PPARγ and LxRs, which are associated with M2 polarization [62], were downregulated by DGLA after 24 h of incubation. This suggests that the effects of DGLA found in this work are unlikely associated with M1 to M2 macrophage polarization but rather with the alleviation of the inflammatory M1 phenotype. This raises the possibility that the immunomodulatory activities of DGLA shown in this work are associated with the enhancement of PGE1. Similarly, it has recently been shown PGE1 is involved in the antiatherogenic actions of DGLA, implicated in the inhibition of macrophage foam cell formation [3]. Detailed eicosanoid profiling is, however, necessary to further evaluate the DGLA-mediated effects.

## 5. Conclusions

Here, we demonstrated that DGLA administration in several forms modulated the immune response in RAW264.7 macrophages. While this work was confined to a cell model, the results further support the anti-inflammatory and immunomodulatory potential of the *L. incisa*-derived DGLA, previously demonstrated in zebrafish feeding trials [23,63]. Additional studies are required to translate these findings to the use of DGLA as feed and health-promoting food supplements for conditions and pathologies that are caused by imbalance in the immune system.

## Supplementary Materials

The following are available online at https://www.mdpi.com/2072-6643/12/9/2892/s1: Table S1: Primers used in this study; Figure S1. Cytokine and NO levels determined in culture supernatants of un-stimulated RAW264.7 macrophages; Figure S2. Fatty acid composition of polar lipids of DGLA-treated RAW264.7 macrophages; Figure S3. Fatty acid composition of microalgal biomass used for production of ethyl esters.
