# Supplementary material for: DGLA from the Microalga Lobosphaera Incsa P127 Modulates Inflammatory Response, Inhibits iNOS Expression and Alleviates NO Secretion in RAW264.7 Murine Macrophages
Source: Nutrients. 2020 Sep 22;12(9):2892. doi: 10.3390/nu12092892 (PMC7551185; doi:10.3390/nu12092892)
Supplement: Supplementary file 1 [file nutrients-12-02892-s001.pdf]

Supplementary material

Table S1: Primers used in the quantitative real-time PCR

Gene	Sequence 5′-3′	GeneBank Accession no.
Arginase	F-AACACGGCAGTGGCTTTAACC	U51805.1
	R-GGTTTTCATCTGGCGCATTC	
Cyclooxygenase-2	F-GTGCCTGGTCTGATGATGTATG	NM_011198.4
	R-TGAGTCTGCTGGTTTGGAATAG	
Fads1Δ5	F-CCAGATTGAACACCACCTCTT	XM_006527438.2
	R-GACTCATACTTGATGCCGTACTT	
Hprt1	F-CAGGGATTTGAATCACGTTTGT	NM_013556.2
	R-TCAACAGGACTCCTCGTATTTG	
iNOS	F-GGAATCTTGGAGCGAGTTGT	NM_010927.4
	R-CCTCTTGTCTTTGACCCAGTAG	
Interleukin 6	F-TGGAGTCACAGAAGGAGTGGCTAAG	NM_031168.2
	R-TCTGACCACAGTGAGGAATGTCCAC	
LXRα	F-TCAAGGGAGCACGCTATGTC	NM_013839.4
	R-TTCCTCTTCTTGCCGCTTC	
NF-kB1	F-AGACATCCTTCCGCAAACTC	XM_006501106.3
	R-TAGGTCCTTCCTGCCCATAA	
PGDS	F-GCAGAACACATGCAAACTGTAG	NM_019455.4
	R-CACTAGGAGCAGTCCCATTTC	
PGES	F-GCAACGACATGGAGACAATCTA	XM_030251932.1
	R-TGTGAGGACAACGAGGAAATG	
PPARγ	F-GCCATTGAGTGCCGAGTC	XM_017321456.2
	R-TGTGGATCCGGCAGTTAAG	

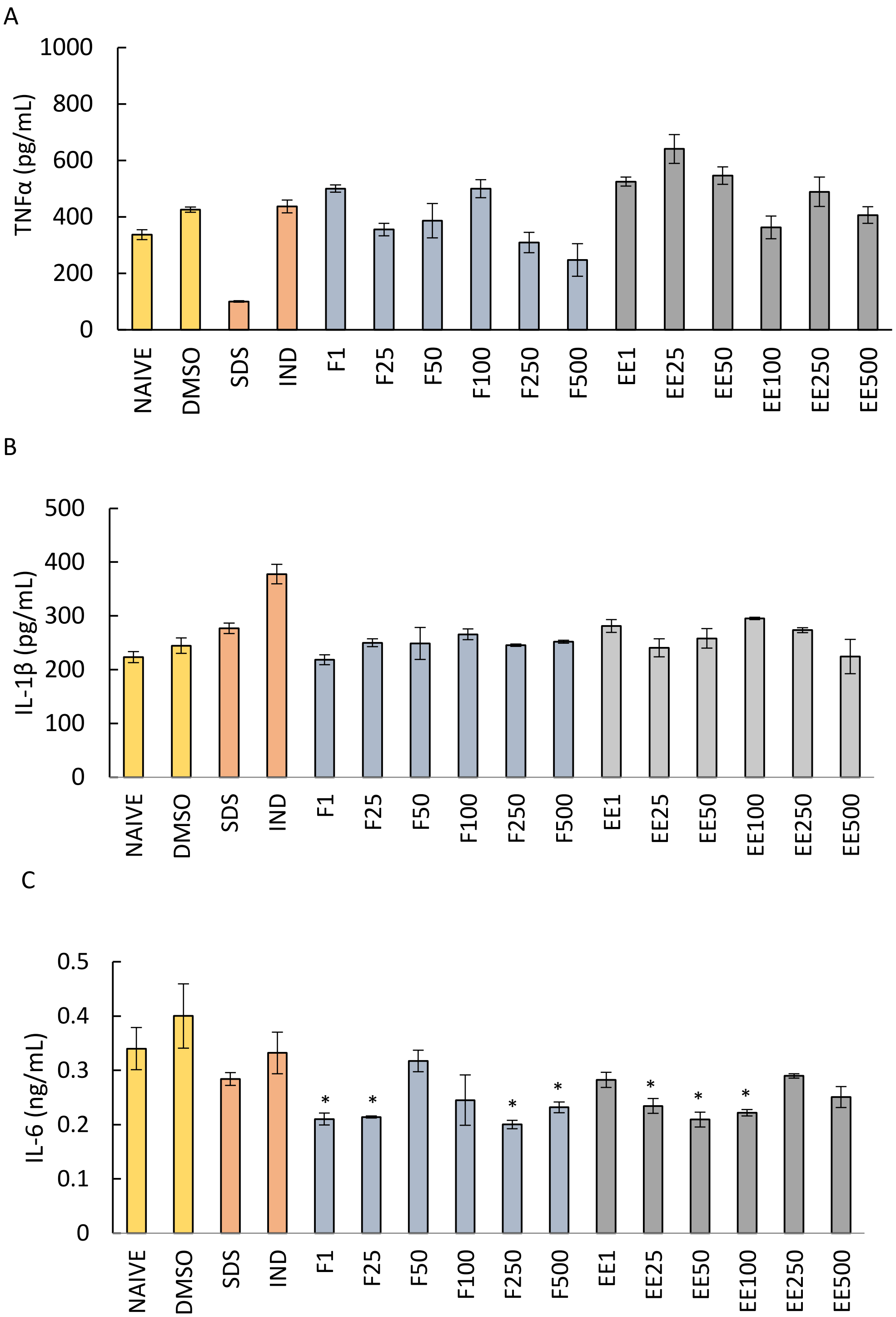
A
TNFα (pg/mL)
0
200
400
600
800
1000
NAIVE
DMSO
SDS
IND
F1
F25
F50
F100
F250
F500
EE1
EE25
EE50
EE100
EE250
EE500
B
IL-1β (pg/mL)
0
100
200
300
400
500
NAIVE
DMSO
SDS
IND
F1
F25
F50
F100
F250
F500
EE1
EE25
EE50
EE100
EE250
EE500
C
IL-6 (ng/mL)
0
0.1
0.2
0.3
0.4
0.5
*
NAIVE
DMSO
SDS
IND
F1
F25
F50
F100
F250
F500
EE1
EE25
EE50
EE100
EE250
EE500

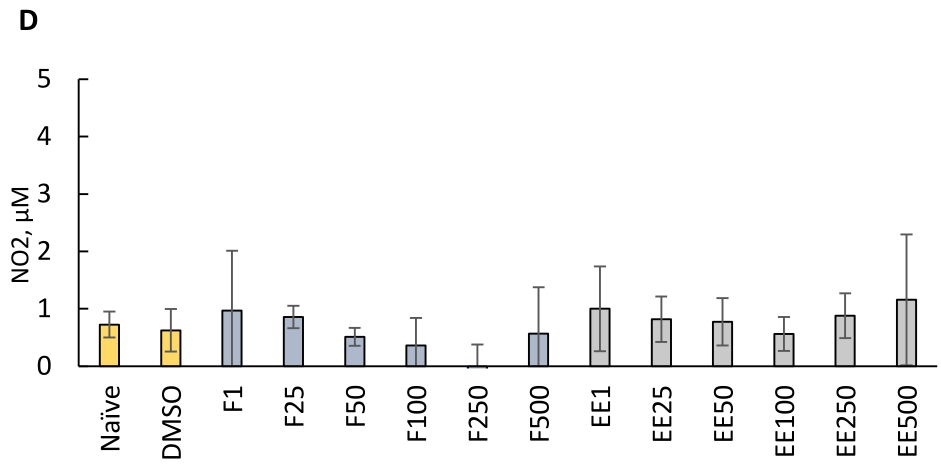

Figure S1: Basal levels of TNFα (A), IL-1β (B), IL-6 (C) and NO (D) detected in the culture supernatants of unstimulated RAW264.7 macrophages. The cells were treated with the indicated concentrations (from 1 to 500 µM) of DGLA administered as free acid (F) and ethyl ester (EE) forms for 24 h. Then, the spent media was collected, and the levels of cytokines and NO were quantified. * denotes a significant difference compared to vehicle control (Student's t-test, p>0.05, n=3)

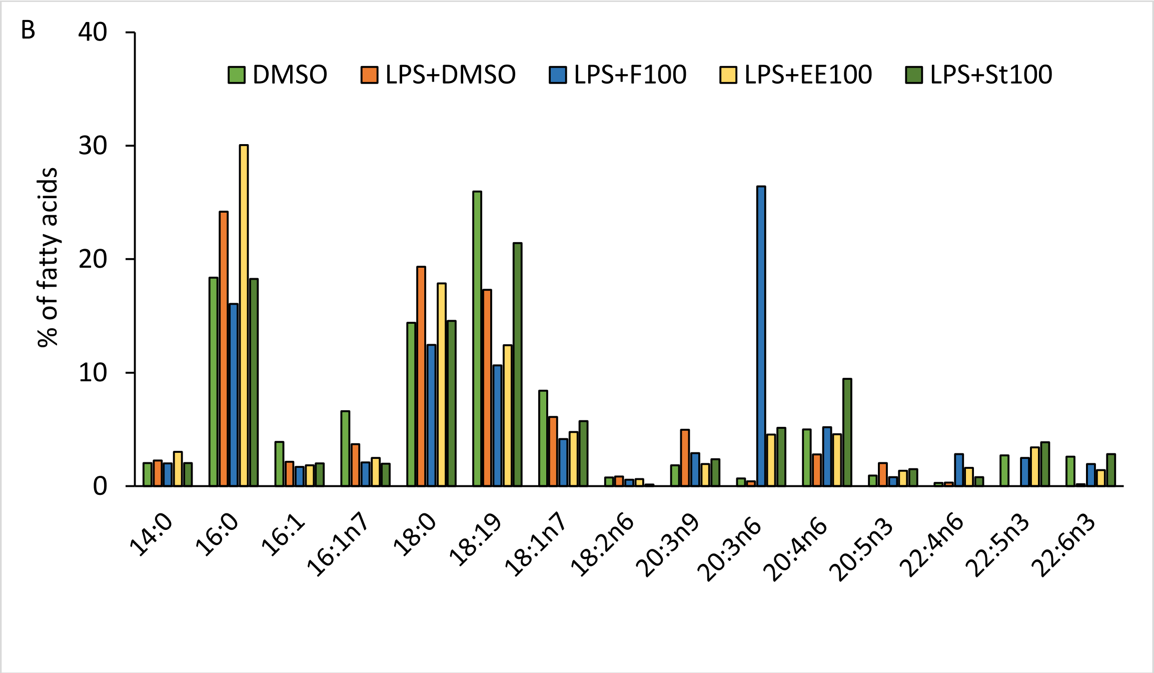

Figure S2: Fatty acid composition of polar lipids of RAW264.7 macrophages under different treatments without (DMSO) or with LPS (100 ng/ml) stimulation. Total lipids were extracted after 24h of incubation with different DGLA forms (indicated) from approximately 10^7 cells; polar lipids were isolated from total lipids on silica-gel cartridges (see Materials and Methods). The fatty acid profile of polar lipids was determined by GC-FID as fatty acid methyl esters. Results from the representative experiment are shown as an average of two determinations.

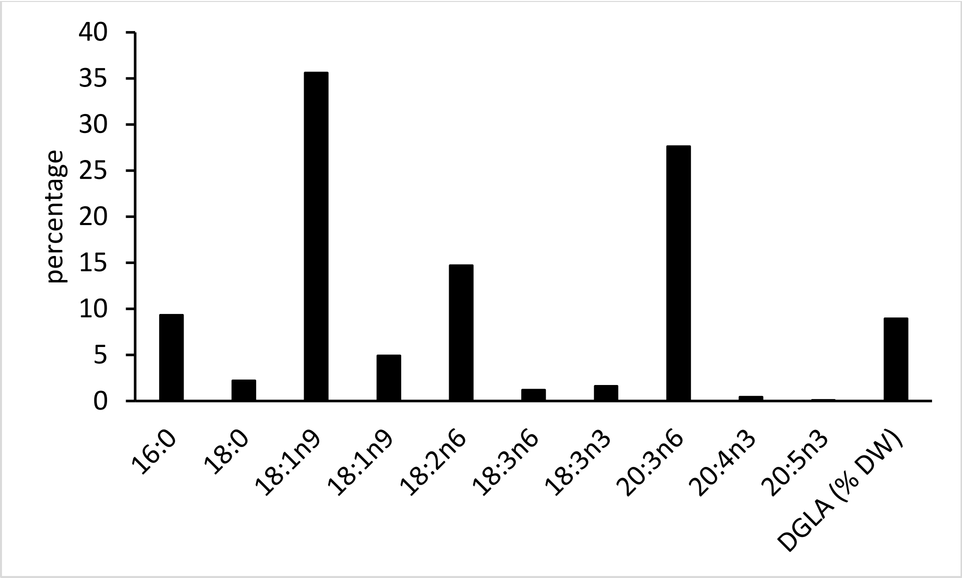

Figure S3: Fatty acid composition and DGLA content of *L. incisa* P127 biomass. The microalga was cultivated under nitrogen starvation conditions to induce DGLA accumulation in triacylglycerols, as indicated in the Methods and Materials section. Fatty acid composition is presented as percentage of total fatty acids, n=2
